# Global burden of young-onset Alzheimer's disease and other dementias: a secondary analysis of the global burden of disease study, 2019

**DOI:** 10.1590/1980-5764-DN-2024-0134

**Published:** 2024-08-19

**Authors:** Diego Fernando Rojas-Gualdrón, Manuela Sánchez Henao, Carlos Alberto Uribe Zuluaga, Alejandro Espinosa Henao, Clara Angela Gómez Henck

**Affiliations:** 1 Universidad CES, Facultad de Medicina, Medellin, Antioquia, Colombia.

**Keywords:** Dementia, Alzheimer Disease, Global Burden of Disease, Health Status Disparities, Disability Studies, Mortality, Demência, Doença de Alzheimer, Carga Global da Doença, Disparidades nos Níveis de Saúde, Estudos sobre Deficiências, Mortalidade

## Abstract

The aging of the world population has led to an increase in the epidemiology and burden of Alzheimer's disease and other dementias.

**Objective:**

To describe the global burden of young-onset Alzheimer's disease and other dementias by world region and income through a secondary analysis of the Global Burden of Disease Study 2019.

**Methods:**

This is a descriptive cross-sectional ecological study. Data by sex and five-year age groups from 40 to 64 years were extracted from the Global Burden of Disease Study results tool. We performed a descriptive analysis of prevalence, incidence, deaths, disability-adjusted life years, years of life lost, and years lived with disability.

**Results:**

In 2019, young-onset Alzheimer's disease and other dementias presented a prevalence of 2.67 cases and an incidence of 0.44 per 1,000 inhabitants globally. It carried a significant burden, resulting in 1.16 disability-adjusted life years per 1,000 inhabitants, primarily due to years of life lost, and to a lesser extent due to years lived with disability. East Asia & the Pacific, Latin America & the Caribbean, and North America are the most affected regions. Burden rates are consistently higher among women; no gradient was observed by country income. Smoking was the most relevant risk factor, presenting a broad difference by country income level.

**Conclusion:**

The global burden of young-onset Alzheimer's disease and other dementias may reshape healthcare requirements and the societal impact of dementias, and its understanding is relevant to inform decisions related to service offerings and research agendas.

## RESUMO.

O envelhecimento da população mundial tem levado a um aumento da epidemiologia e da carga da Doença de Alzheimer e outras Demências.

**Objetivo:**

Descrever a Carga Global da Doença de Alzheimer de Início Precoce e outras Demências (DAIPoD) por região do mundo e nível de renda por meio de uma análise secundária do Estudo Global de Carga de Doenças (*Global Burden of Disease Study* – GBD), 2019.

**Métodos:**

Estudo ecológico transversal descritivo. Os dados de cinco anos por sexo e faixa etária de 40 a 64 anos foram extraídos da ferramenta de resultados do GBD. Realizou-se uma análise descritiva de prevalência, incidência, óbitos, Anos de Vida Ajustados por Incapacidade (*Disability Adjusted Life Years* – DALY), Anos de Vida Perdidos *(Years of Life Lost* – YLL) e Anos Vividos com Incapacidade (*Years Lived with Disability* – YLD).

**Resultados:**

Em 2019, a DAIPoD apresentou uma prevalência de 2,67 casos e uma incidência de 0,44 casos por 1.000. Foi observada uma carga significativa, resultando em 1,16 DALY por 1.000, principalmente devido aos YLL e, em menor grau, aos YLDs. As regiões mais afetadas foram o Leste Asiático e Pacífico, a América Latina e Caribe, e a América do Norte. As taxas de carga são consistentemente mais elevadas entre as mulheres; não foi observado um gradiente por nível de renda dos países. O tabagismo foi o fator de risco mais relevante, apresentando uma ampla diferença por nível de renda do país.

**Conclusão:**

A carga global do DAIPoD pode remodelar as necessidades de saúde e o impacto social das demências, e sua compreensão é relevante para informar decisões relacionadas à oferta de serviços e agendas de pesquisa.

## INTRODUCTION

According to the Global Burden of Disease Study (GBD) 2019^1^, the aging of the world population has led to an increase in the epidemiology and burden of Alzheimer's Disease and other Dementias (ADoD). The regions most affected by mortality are Asia-Pacific and Sub-Saharan Africa, while Latin America is the most impacted regarding Disability-Adjusted Life Years (DALY). Notably, people living in areas with a high sociodemographic index and women exhibit a higher impact. Despite the concentration of the burden among those over 65, there is a growing interest in Young-Onset Alzheimer's Disease and other Dementias (YOADoD), with onset in individuals younger than 65 years^2^.

Clinical evidence has highlighted some significant differences according to age at onset. The heterogeneous etiology of YOADoD leads to a broad spectrum of symptoms with a more aggressive disease progression^3^, and because individuals under 65 often have more active economic roles in society, it results in a different impact on disability. A qualitative study by Bakker et al. identified specific needs related to employment, finances, and housing^4^. On the other hand, survival tends to be longer for early-onset patients^5^, which, combined with its disability impact, may result in a differentiated global burden landscape.

Understanding the prevalence, incidence, mortality, and disability of YOADoD is crucial for evidence-based healthcare planning, priority setting, and resource allocation^6^. Some reviews^7,8^ and registry-based studies^9–12^ have provided valuable insights drawn from original studies and routinely collected data from specific countries. However, a clear depiction of the global burden is lacking. Given its characteristics and geographic scope, GBD is a relevant source to contribute to burden understanding.

This study aimed to describe the global burden of YOADoD by world region and income through a secondary data analysis of the GBD 2019.

## METHODS

As the analysis herein uses aggregated publicly available data, this study did not require approval by an ethics committee.

### Study design and settings

This is a descriptive cross-sectional ecological study, based on the estimations of the GBD 2019 — "a systematic scientific assessment of published, publicly available, and contributed data on incidence, prevalence, and mortality for a mutually exclusive and collectively exhaustive list of diseases and injuries"^13^. All data, software code, and methodological manuals are publicly available at the GBD site (https://www.healthdata.org/research-analysis/gbd).

### Participants

Data were extracted by sex and five-year age groups from 40 to 64 years. Aggregated estimations were obtained and analyzed according to the classification of the World Bank Atlas methodology^14^ by world region: East Asia & the Pacific, Europe & Central Asia, Latin America & the Caribbean, Middle East & North Africa, North America, South Asia, and Sub-Saharan Africa; and by country income groups such as high-income countries (HIC), upper-middle-income countries (UMIC), lower-middle-income countries (LMIC), and low-income countries (LIC).

### Variables and data sources

The publicly available estimations of population and cases by prevalence, incidence, deaths, DALY, Years of Life Lost (YLL), and Years Lived with Disability (YLD) were extracted from the GBD results tool^15^. The methods to estimate rates can be consulted on the GBD results tool website. Age-specific YOADoD rates were estimated and presented per 1,000 inhabitants. Data on deaths, DALY, YLL, and YLD associated with risk factors was also retrieved and analyzed. The GBD 2019 included three risk factors for ADoD, as according to their analyses, sufficient evidence exists for high body mass index, high fasting plasma glucose, and smoking^16^.

### Statistical methods

The burden of YOADoD by world region and income group is described by presenting the prevalence, incidence, DALY, YLL, YLD, and mortality rates (per 1,000 inhabitants). The differences in the epidemiology and burden of disease by world region and income group were analyzed by comparing each subgroup rate with the global rate.

Sex differences (female-to-male) in the burden of YOADoD were explored using the typology proposed by Blakely et al.^17^. This analytical framework jointly considers the population (P) rate (i.e., both sexes), the relative (R) difference (i.e., rate ratio), and the absolute (A) difference (i.e., rate difference) to obtain a PAR (Population-Relative-Absolute) profile to compare to the overall population. An arrow is used to depict if a subgroup has P, A, or R higher (↑), lower (↓), or similar (-) to the global estimation. Highly desirable states (dark green) must include lower P (P↓) and lower A (A↓), irrespective of the R rate. Desirable states (light green) must include lower P (P↓) and lower or similar A (A↓ or A-), irrespective of R. Undesirable states (red) include higher P (P↑) irrespective of A and R, or a similar P (P-) with higher sex differences in A (A↑) or R (R↑). The remaining profiles are classified as equivocal (yellow).

Lastly, the burden of YOADoD associated with the three reported risk ratios is described by presenting the DALY, YLL, YLD, and mortality rates (per 100,000 inhabitants) by world region and income group.

## RESULTS

**Figure 1:**
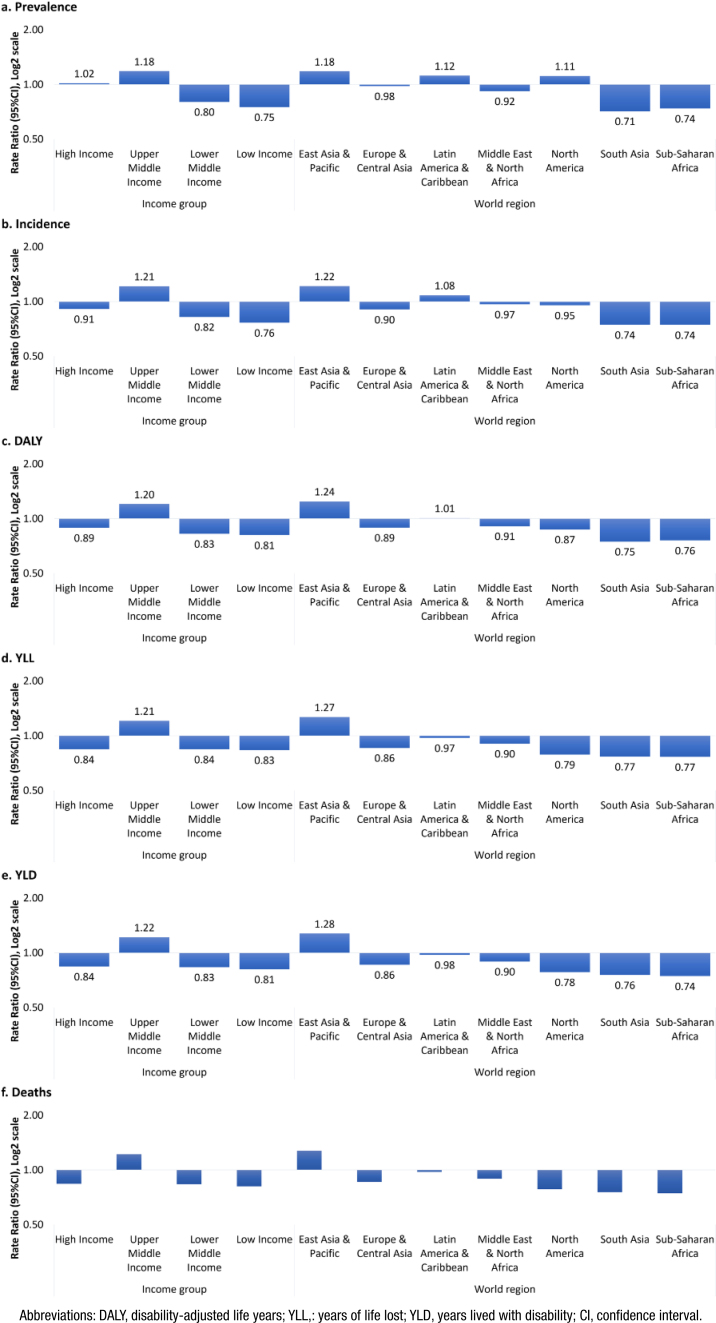
World region and income level differences in the epidemiology and burden of young-onset (40–64 years) Alzheimer's disease and other dementias, both sexes, 2019.

During 2019, YOADoD prevalence and incidence rates were 2.67 and 0.44 per 1,000 inhabitants, respectively. Regarding its burden, the DALY rate was 1.16, which can be decomposed into 0.84 due to YLL and 0.32 due to YLD per 1,000 inhabitants. The death rate was 0.27 per 1,000 inhabitants. Table 1 presents the estimated rates by sex, income groups, and world regions, and Figure 1 shows the estimated rate ratios.

**Table 1:** Epidemiology and burden of young-onset (40–64 years) Alzheimer's disease and other dementias, by world regions, income level groups, and sex, 2019.

Sex		World regions								Income level			
		Global	East Asia & the Pacific	Europe & Central Asia	Latin America & the Caribbean	Middle East & North Africa	North America	South Asia	Sub-Saharan Africa	HIC	UMIC	LMIC	LIC
Prevalence rate													
	B	2.67	**3.15**	2.62	2.99	2.45	2.98	1.90	1.97	2.72	**3.16**	2.14	2.00
	F	2.88	**3.39**	2.89	3.09	2.63	2.98	2.18	2.11	2.89	**3.37**	2.40	2.14
	M	2.45	2.92	2.33	2.87	2.30	**2.97**	1.63	1.83	2.55	**2.95**	1.88	1.86
Incidence rate													
	B	0.44	**0.53**	0.39	0.47	0.42	0.42	0.33	0.32	0.40	**0.53**	0.36	0.33
	F	0.47	**0.57**	0.44	0.49	0.45	0.41	0.38	0.36	0.42	**0.57**	0.41	0.37
	M	0.40	**0.49**	0.35	0.46	0.40	0.43	0.27	0.29	0.37	**0.49**	0.31	0.30
DALY rate													
	B	1.16	**1.44**	1.03	1.17	1.06	1.01	0.87	0.88	1.03	**1.40**	0.96	0.94
	F	1.23	**1.55**	1.11	1.23	1.14	1.01	0.93	0.91	1.05	**1.49**	1.04	0.98
	M	1.09	**1.34**	0.94	1.14	0.96	1.02	0.80	0.84	0.99	**1.30**	0.88	0.88
YLL rate													
	B	0.84	**1.06**	0.72	0.81	0.76	0.66	0.64	0.64	0.70	**1.01**	0.70	0.70
	F	0.89	**1.14**	0.76	0.85	0.82	0.65	0.68	0.66	0.71	**1.08**	0.75	0.72
	M	0.79	**0.98**	0.65	0.79	0.68	0.66	0.60	0.62	0.68	**0.94**	0.66	0.66
YLD rate													
	B	0.32	**0.38**	0.32	0.36	0.30	0.35	0.23	0.24	0.33	**0.38**	0.25	0.24
	F	0.35	**0.41**	0.35	0.37	0.31	0.35	0.25	0.26	0.34	**0.41**	0.28	0.26
	M	0.30	**0.36**	0.28	0.35	0.28	0.35	0.20	0.22	0.31	**0.36**	0.23	0.23
Mortality rate													
	B	0.27	**0.34**	0.23	0.26	0.24	0.21	0.20	0.20	0.22	**0.33**	0.22	0.22
	F	0.28	**0.37**	0.24	0.27	0.26	0.21	0.21	0.21	0.23	**0.35**	0.24	0.23
	M	0.25	**0.31**	0.21	0.25	0.21	0.21	0.19	0.19	0.22	**0.30**	0.21	0.20

Abbreviations: HIC, high-income countries; UMIC, upper-middle-income countries; LMIC, lower-middle-income countries; LIC, low-income countries; B, both sexes; F, females; M, males; DALY, disability-adjusted life years; YLL, years of life lost; YLD, years lived with disability.

Notes: All rates are per 1,000 inhabitants; bolded numbers refer to the highest rates.

### Burden of YOADoD by world region

For both sexes’ prevalence and incidence rates, East Asia & the Pacific region showed results that surpassed global averages by 18% and 22%, respectively. Latin America & the Caribbean (PRR=1.12) and North America (PRR=1.11) also presented prevalences exceeding the global rate; however, the incidence was only higher in Latin America (IRR=1.08). East Asia & the Pacific was the region with the highest disease burden, surpassing the global mortality rate by 28% and the DALY rate by 24%. Latin America was the second-highest burdened region, with mortality RR and DALY RR of 0.98 and 1.01, respectively.

### Burden of YOADoD by income group

UMIC presented the highest rates for all burden metrics. Moreover, it was the only group with rates higher than the global estimate, except for prevalence, where HIC also showed a higher prevalence of 2%. Differences between LIC, LMIC, and HIC are minimal for mortality and DALY rates. LIC showed the lowest prevalence and incidence rates.

### Burden of YOADoD by sex differences

**Figure 2:**
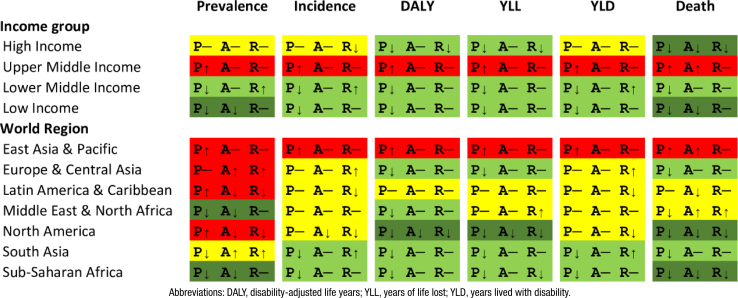
Sex differences in the epidemiology and burden of young-onset (40–64 years) Alzheimer's disease and other dementias, by world region and income group, 2019.

Notes: P: population rate (both sexes). A: absolute rate difference (female-male). R: relative rate difference (female/male). ↓: Lower than; ↑: Higher than; or −: similar (to the global rate). Dark green: highly desirable. Light green: desirable. Yellow: equivocal. Red: undesirable.

A sex disparity exists in burden metrics, with women generally experiencing less favorable outcomes (Figure 2). Globally, for every 1,000 inhabitants, women exhibit additional 0.43 prevalent cases (PRR=1.18), 0.07 incident cases (IRR=1.19), and 0.15 DALY (RR=1.13); additionally, women presented 0.34 more deaths (RR=1.13) than men per 100,000 inhabitants. Compared to the global profile, a less favorable sex disparity scenario is observed among UMIC and inhabitants from East Asia & the Pacific (Figure 2).

### Risk factors for YOADoD

**Figure 3:**
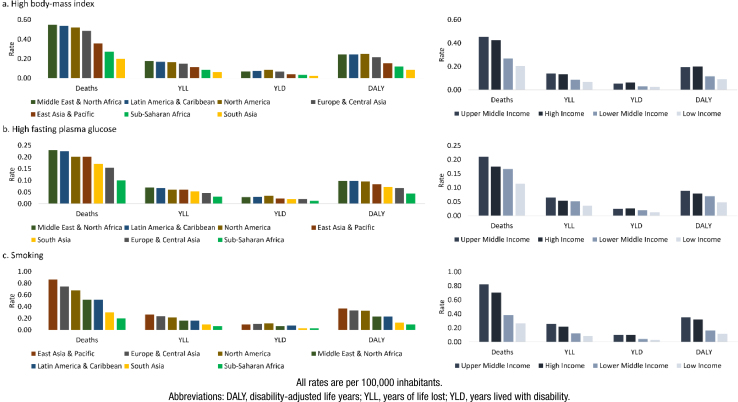
Risk factors for the burden of young-onset (40-64 years) Alzheimer's disease and other dementias, by world region and income group, both sexes, 2019.

Globally, the impact of smoking surpassed that of other risk factors, with rates of 0.62 for mortality, 0.19 for YLL, 0.08 for YLD, and 0.27 for DALY (Figure 3). For high body mass index and high fasting glucose, the Middle East & North Africa, Latin America & the Caribbean, and North America had the highest associated burden rates. Regarding country income, UMIC and HIC had the highest risk factor-associated burden rates.

## DISCUSSION

This study aimed to describe the epidemiology and burden of YOADoD worldwide through a secondary analysis of the GBD 2019. YOADoD had a prevalence of 2.67 cases and an incidence of 0.44 cases per 1,000 inhabitants globally. It carries a significant disease burden, resulting in 1.16 DALY per 1,000 inhabitants. Most of these DALY are due to YLL and, to a lesser extent, due to YLD. East Asia & the Pacific, Latin America & the Caribbean, and North America are the regions most affected; however, the burden in North America is lower relative to its prevalence and incidence. Burden rates are consistently higher among women; no gradient was observed by country income. Smoking was the most relevant risk factor, presenting a broad difference between HIC-UMIC and LMIC-LIC.

A previous study analyzed the burden of ADoD (≥40 years) and its changes from 1990 to 2019^1^. The authors found a significant increase in the incidence and prevalence of ADoD. Their regional analysis of the 2019 data shows that Eastern European and East Asian countries had the highest incidence, while the Sub-Saharan Africa and Latin America regions had the lowest. This regional distribution contrasts with our findings on YOADoD, in which higher incidence was observed for Latin America & the Caribbean, the Middle East & North Africa, and North America. On the other hand, a positive correlation was described between sociodemographic index and the prevalence and incidence of ADoD, while we did not find a correlation with country income for YOADoD; and UMIC tended to have higher rates in our YOADoD analysis.

Regarding burden, the highest ADoD DALY rates were described for East Asia and Eastern Europe^1^, while the highest rates were observed for YOADoD in East Asia and Latin America. Regarding deaths, the mortality rate was higher in East Asia & the Pacific for YOADoD, while for ADoD, a higher mortality rate was observed in Europe & Central Asia^1^. Previous studies have highlighted a higher mortality risk among people with young onset dementia^18^.

Sex differences with a higher burden among women were observed in the analysis of YOADoD and ADoD, although the disparity was more pronounced in the latter. A systematic review by Huque et al.^19^ found that country-level differences in life expectancy and education explained sex differences in ADoD. However, in that review, including 205 studies, the authors found no differences in age-specific incidence rates, only in the prevalence. Genetics, lifestyle, and sex-specific risk factors have been explored at the individual level as explanatory factors for sex differences in ADoD^20–22^.

The wealth advantage of HIC, compared to UMIC, favoring environmental risk factors, healthcare, healthy life expectancy, and education, may also explain their lower prevalence and burden^23^. On the other hand, the low burden observed in LIC may be explained by aging patterns, differences in environmental and genetic factors, or healthcare access and quality. The coverage and quality of registries likely play a role in the reported statistics^24^. Particularly, data on YOADoD is scarce among LIC and LMIC, as early detection is highly dependent on healthcare characteristics. However, it is not possible to determine if the global distribution of those factors explains the global differences in the burden of the disease.

In two separate systematic reviews, Hendrick et al. estimated a prevalence of YOADoD of 1.19 per 1,000 inhabitants^7^ and an incidence of 1.1 per 1,000 inhabitants^8^, including data from 30 to 64 years of age. The estimated prevalence was lower, and the incidence was higher than those obtained from the GBD 2019. The GBD is focused on estimating disease burden and providing prevalences and incidences for specific years. However, as they synthesized and discussed findings from individual studies, those systematic reviews provided valuable information that was lacking in the sources of the GBD.

The main limitation of this study is that all estimated rates, except for incidence, lack data on people older than 65 years living with YOADoD. As the GBD is a cross-sectional study, it is impossible to disaggregate data according to age at onset. However, the estimations presented are a relevant contribution to the evidence of YOADoD that complements efforts to synthesize and meta-analyze individual studies. Additionally, this study has the intrinsic limitations of an ecological investigation, and, as a secondary data analysis study, it inherits the limitations of a source investigation.

YOADoD care is a long journey challenged by significant economic burdens, caregiver distress, and increasing service demands^25,26^. The global burden of YOADoD may reshape healthcare requirements and the societal impact of dementias, and its understanding is relevant to inform decisions related to evidence-based healthcare planning, priority setting, and resource allocation, and to build research agendas.
